# Dissecting the tRNA Fragment tRF3E–Nucleolin Interaction: Implications in Breast Cancer

**DOI:** 10.3390/biom15071054

**Published:** 2025-07-21

**Authors:** Maurizio Falconi, Junbiao Wang, Andrea Costamagna, Mara Giangrossi, Sunday Segun Alimi, Emilia Turco, Massimo Bramucci, Luana Quassinti, Rossana Petrilli, Michela Buccioni, Gabriella Marucci, Augusto Amici, Paola Defilippi, Roberta Galeazzi, Cristina Marchini

**Affiliations:** 1 School of Biosciences and Veterinary Medicine, University of Camerino, 62032 Camerino, Italy; junbiao.wang@unicam.it (J.W.); mara.giangrossi@unicam.it (M.G.); sundaysegun.alimi@studenti.unicam.it (S.S.A.); rossana.petrilli@unicam.it (R.P.); augusto.amici@unicam.it (A.A.); 2 Department of Molecular Biotechnology and Health Sciences, University of Turin, 10126 Turin, Italy; a.costamagna@unito.it (A.C.); emilia.turco@unito.it (E.T.); paola.defilippi@unito.it (P.D.); 3 School of Pharmacy, University of Camerino, 62032 Camerino, Italy; massimo.bramucci@unicam.it (M.B.); luana.quassinti@unicam.it (L.Q.); michela.buccioni@unicam.it (M.B.); gabriella.marucci@unicam.it (G.M.); 4 Department of Life and Environmental Sciences, Marche Polytechnic University, 60131 Ancona, Italy; r.galeazzi@staff.univpm.it

**Keywords:** nucleolin, tRNA fragments, RNA–protein interaction, breast cancer

## Abstract

Nucleolin (NCL), an RNA-binding protein which regulates critical cellular processes, is frequently dysregulated in human cancers, including breast cancer, making it an attractive therapeutic target. However, molecular details of the RNA-NCL interaction have not been investigated yet. A tRNA fragment named tRF3E, displaying tumor suppressor roles in breast cancer, was found to bind NCL with high affinity displacing NCL-controlled transcripts. Here, we investigated the determinants and cooperativity of tRF3E-NCL interaction by Electrophoretic Mobility Shift Assays and in silico docking analysis, using wild-type or mutated tRF3E. We found that NCL, through its RNA-binding domains (RBD1–2 and RBD3–4), binds simultaneously two tRF3E molecules, giving rise to an energetically favored complex. Instead, a mutant form of tRF3E (M19–24), in which the NCL recognition element in position 19–24 has been disrupted, contacts NCL exclusively at RBD3–4, causing the loss of cooperativity among RBDs. Importantly, when expressed in MCF7 breast cancer cells, tRF3E significantly reduced cell proliferation and colony formation, confirming its role as tumor suppressor, but tRF3E functional properties were lost when the 19–24 motif was mutated, suggesting that cooperativity among multiple domains is required for the NCL-mediated tRF3E antitumor function. This study sheds light on the dynamic of RNA-NCL interaction and lays the foundations for using tRF3E as a promising NCL-targeted biodrug candidate.

## 1. Introduction

Nucleolin (NCL) is an evolutionarily conserved RNA-binding protein, highly expressed in the nucleolus, where it plays essential roles in ribosome biogenesis; it is also found in the nucleoplasm, cytoplasm and cell membrane [1]. NCL is a multifunctional protein able to modulate crucial molecular processes such as cell proliferation and survival [2]. The multiple functions of NCL reflect its complex structure. NCL is composed of 710 amino acid residues, and it can be divided into three main structural regions endowed with specific activities. The N- and C-terminal regions are involved in the interaction with other proteins, including components of the pre-rRNA processing complex and ribosomal proteins. The central region of NCL contains four tandem RNA-binding domains (RBS1–2 and RBS3–4) that mediate its interaction with target RNAs, such as ribosomal RNA precursor (pre-rRNA), and permit the post-transcriptional regulation (processing, stability and translation) of recognized messenger RNAs [3]. The NCL recognition element (NRE) is characterized by a G-rich signature motif (U/G)CCCG(A/G) within a stem-loop structure [4,5]. NCL is also involved in pathological processes, particularly cancer and viral infections [6]. Indeed, it is highly expressed and associated with poor prognosis in several different human cancers, including gastric cancer [7], breast cancer [8], non-small-cell lung cancer [9], pancreatic cancer [10,11] and endometrial cancer [12]. The p53 and BCL-2 mRNAs emerge among the NCL-regulated transcripts having a role in cancer. The p53 mRNA translation is inhibited by NCL binding [13,14], whereas BCL-2 mRNA stability is enhanced by the interaction with NCL [15,16]. Given its implication in oncogenic processes, NCL has attracted attention as a druggable target. Various therapeutic molecules have been developed to control NCL activity, such as NCL-directed anticancer aptamers. Particularly, the aptamer AS1411, a 26-mer unmodified guanosine (G)-rich oligonucleotide, is among the synthetic compounds most extensively studied [17] and was tested as an anticancer agent in Phase II clinical trials, showing good therapeutic efficacy and tolerability [18,19].

tRNA-derived RNA fragments (tRFs) represent an important new category of small non-coding RNAs with regulatory roles. A dysregulation of tRFs has been reported in tumor cells, where they may have oncogenic or tumor-suppressor functions. tRFs can operate through different mechanisms [20,21]. They can inhibit gene expression through miRNA-like silencing or modulate translation by displacing eukaryotic initiation factors from mRNAs [22] or by competing with mRNA for ribosome binding [23]. Moreover, tRFs can exert their function by interacting with proteins, in particular RNA-binding proteins. A Cysteine tRNA fragment (5′-tRF^Cys^) was recently found to increase during breast cancer metastatic progression. 5′-tRF^Cys^ promotes the oligomerization of NCL into a transcript-stabilizing ribonucleoprotein complex, protecting these messenger RNAs from degradation [24]. Conversely, a specific set of tRFs induced by hypoxia, with tumor-suppressive and metastasis-suppressive activity, was identified in breast cancer cells by Goodarzi et al. [25]. These tRFs, including a tRF^Glu^, were found to specifically interact with the RNA-binding protein Y-box Binding Protein 1 (YBX1), leading to a competitive displacement of endogenous oncogenic transcripts from YBX1, thereby promoting their destabilization.

In a recent study, we identified a 32 nt tRF (named tRF3E), derived from mature tRNA^Glu^, which is downregulated in breast cancer. tRF3E acts as a tumor-suppressor and operates through a mechanism that is dependent on the physical interaction with NCL [26]. Indeed, tRF3E, thanks to its binding properties, is potentially able to displace specific transcripts controlled by NCL. In particular, we provided evidence that tRF3E can compete with p53 mRNA for NCL binding. As a consequence, p53 mRNA is released by NCL and translated, leading to a reduction in cell proliferation [26]. According to the determinants required for the formation of stable RNA-NCL complexes, tRF3E bears two nucleotide stretches matching the identified NRE. The high binding affinity of tRF3E for NCL is demonstrated by the low dissociation constant (K_D_ ≅ 120 nM) of the NCL-tRF3E complex, as calculated by Electrophoretic Mobility Shift Assays (EMSAs) [26]. Such a K_D_ value is even lower than that estimated for the binding sites B1 and B2 of pre-rRNA, a well-known natural target of NCL [4,5]. Moreover, NCL protein can form a complex containing two molecules of tRF3E that simultaneously occupy RBS1–2 and RBS3–4 [26]. Thus, tRF3E exerts tumor-suppressor functions by competitive displacement of NCL-regulated transcripts.

Here, we analyzed in depth the critical determinants of the tRF3E-NCL complex and the functional role of the binding cooperativity, aiming to validate tRF3E as an NCL natural modulator capable of interfering with cell oncogenic behavior. tRF3E might represent a promising NCL-targeted biodrug against breast cancer.

## 2. Materials and Methods

### 2.1. General Procedures

**Figure 1:**
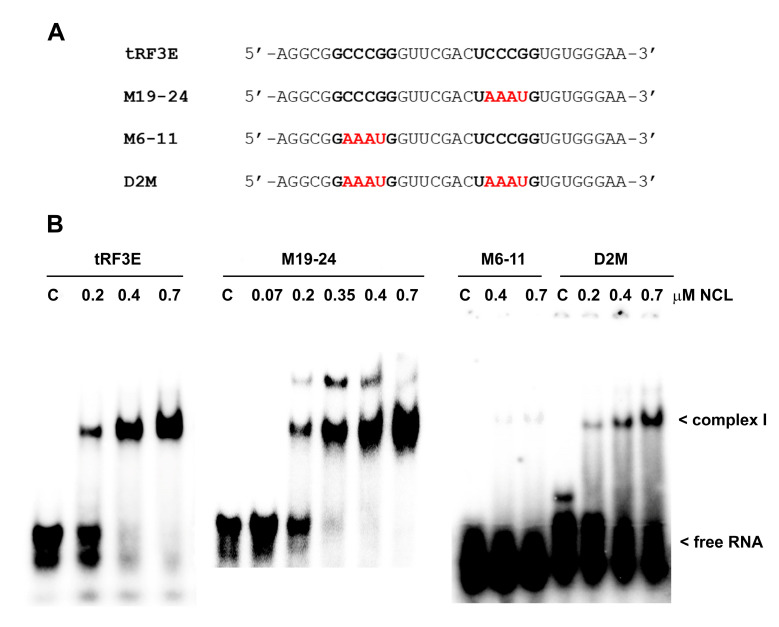
Comparative interaction of NCL with wild-type tRF3E and its mutants by EMSA. (**A**). The sequences of wt tRF3E and its mutants in the (U/G)CCCG(A/G) consensus motif recognized by NCL are reported. Nucleotides on tRF3E matching the NCL binding site are in black (bold) whereas mutated nucleotides are in red. (**B**). EMSA was carried out as described in Section 2, incubating 2 pmol of [^32^P]-labeled RNA oligonucleotides with the indicated concentrations of purified NCL. “C” is the control sample in absence of protein and the electrophoretic migration of free RNA and of RNA-NCL complex I are indicated. K_D_ values, reported in Section 3, were estimated by Molecular Imager quantification of radioactivity associated with bound tRF3E and expressed as the minimal concentration of protein required to retard 50% of the total RNA probe.

DNA and RNA samples were quantified with NanoDrop (Thermo Fisher Scientific, Waltham, MA, USA). RNA oligonucleotide wild-type (wt) tRF3E and its mutants (shown in Figure 1A) were [^32^P]-labeled using the T4 polynucleotide kinase. Radioactive bands on solid supports were detected and quantified by Molecular Imager (model FX; Bio-Rad, Hercules, CA, USA). Purified human NCL was provided by MyBioSource (San Diego, CA, USA). This protein is missing the N-terminal region, and its molecular mass is 55 versus 70 kDa of wild-type protein. NCL is expressed with a −6× His tag at N-terminus.

### 2.2. Electroblotting

MCF-7-tRF3E and MCF-7-M19–24 cells were left untreated (control) or induced with 1 µg/mL doxycycline for different times (24 h, 48 h, 72 h and 168 h); then, RNA was extracted from each cell line, using EuroGOLD Trifast^TM^ Kit (Euroclone, Pero (MI), Italy) following the manufacturer’s instructions, and quantified. The RNA samples were resuspended in loading buffer (95% formamide, EDTA 10 mM, bromophenol-blu and xylene cyanol 0.01%), denaturated at 65 °C for 5 min, loaded on 8% PAGE-urea gel in Tris-borate EDTA buffer 0.5 X and transferred to a neutral nylon Hybond-N membrane (Amersham, Little Chalfont, UK) by semidry electroblotting (Trans-Blot Turbo Transfer System; Bio-Rad, Hercules, CA, USA) at 0.3 A for 30 min. The membrane was hybridized with a 20 mer [^32^P]-labeled tRF3E probe (5′-CACCGGGAGTCGAACCCGGGCCGCC-3′) at 56 °C, essentially as described by Sambrook and Russell [27].

### 2.3. Electrophoretic Mobility Shift Assay (EMSA)

EMSA was made in 15 µL of Gel Retardation buffer (20 mM Tris-HCl, pH 7.5, 50 mM KCl, 10% glycerol, 0.3 mg/mL bovine serum albumin, 0.02% Nonidet P-40) incubating 2 pmoles (unless otherwise stated) of [^32^P]–labeled synthetic RNAs with the indicated amounts of NCL at 20 °C for 30 min. Competitive EMSAs were performed under the same experimental conditions, preincubating the [^32^P]-labeled tRF3E with a fixed concentration of NCL (0.5 µM) for 30 min before adding increasing amounts of unlabeled RNAs (wt tRF3E and M19–24) as indicated. Then, the incubation was prolonged for an additional 30 min. Samples were loaded on native 7% polyacrylamide gel run at 4 °C. To efficiently transfer the NCL protein from the EMSA gel to the polyvinylidene difluoride (PVDF) membrane (Immobilion P, Millipore, Burlington, MA, USA) for subsequent immunodetection, the polyacrylamide gel was incubated for 2 h in 40 mL of a solution containing 300 mM Tris-HCl, pH 8.0 and 1% SDS, to make the protein negatively charged before the electrotransfer. The immunodetection of NCL was achieved with an anti-His tag antibody (His-probe (H-3): sc-8036) from Santa Cruz Biotechnology (Dallas, TX, USA) that was visualized with a peroxidase-conjugated second antibody, as described below in the Western blot paragraph.

### 2.4. Cell Cultures and Generation of MCF-7-tRF3E and MCF-7-M19–24 Cell Lines

SK-BR-3 (estrogen receptor-negative, progesteron receptor-negative and human epidermal growth factor receptor (HER2)-positive) human breast cancer cells were cultured in Dulbecco’s Modified Essential Medium (DMEM, CORNING, Corning, NY, USA) supplemented with 10% fetal bovine serum (FBS, Gibco, Thermo Fisher Scientific, Waltham, MA, USA) and 1% penicillin–streptomycin (Gibco, Thermo Fisher Scientific, Waltham, MA, USA). MCF-7 (estrogen receptor-positive, progesteron receptor-positive and HER2-negative) human breast cancer cells were cultured in RPMI (Roswell Park Memorial Institute; CORNING, Corning, NY, USA) supplemented with 10% FBS (Gibco, Thermo Fisher Scientific, Waltham, MA, USA), 1X L-Glutamine and 1% penicillin–streptomycin (Gibco, Thermo Fisher Scientific, Waltham, MA, USA). MCF-7 cells were used as transduction target cells. In brief, MCF-7-tRF3E, MCF-7-M19–24 and MCF-7 control cells were generated by lentiviral transduction of recombinant pLKO-Tet-On vectors, encoding for wt tRF3E or the mutant form of tRF3E (M19–24) or empty vector, respectively. First, the sequences encoding for wt tRF3E or M19–24 were cloned into Tet-pLKO-puro vector (Lenti Tet-pLKO-puro Addgene Plasmid#21915) [28], using AgeI and EcoRI restriction enzymes, in order to be expressed under the control type III RNA Pol III promoter (H1) regulated by doxycycline in stably transduced cells. For this purpose, the forward and reverse oligonucleotides indicated below were annealed. The row of T at the end of the forward oligonucleotides are recognized as stop signals by the RNA polymerase III.

TRF3E Forward 5′-CCGGAGGCGGCCCGGGTTCGACTCCCGGTGTGGGAATTTTT-3′
TRF3E Reverse 5′-AATTAAAAATTCCCACACCGGGAGTCGAACCCGGGCCGCCT-3′
M19–24 Forward 5′-CCGGAGGCGGCCCGGGTTCGACTAAATGTGTGGGAATTTTT-3′
M19–24 Reverse 5′-AATTAAAAATTCCCACACATTTAGTCGAACCCGGGCCGCCT-3′

Lentiviruses were produced according to the manufacturer’s instructions and used to transduce MCF-7 cells. To select transduced cells, puromycin (Sigma Aldrich/Merck, Darmstadt, Germany) (0.5 µg/mL) was added 24 h after infection with recombinant lentiviral vectors as already described [29]. MCF-7-tRF3E, MCF-7-M19–24 and MCF-7 control cells were maintained in RPMI supplemented with 10% FBS, 1X L-Glutamine, 1% penicillin–streptomycin and 0.5 µg/mL puromycin, in a humidified atmosphere with 5% CO_2_ at 37 °C. Doxycycline hyclate (Sigma Aldrich/Merck, Darmstadt, Germany) (1 µg/mL) was added to complete medium to induce tRF3E or M19–24 expression. Induction medium containing doxycycline was changed every 2 days. MCF-7-tRF3E, MCF-7-M19–24 and MCF-7 (empty) control cells were generated and kindly provided by the laboratory of Prof. P. Defilippi (Department of Molecular Biotechnology and Health Sciences, University of Turin, Turin, Italy). Cells were tested for mycoplasma contamination with negative results.

### 2.5. Cell Viability Assay

Cell viability was evaluated by seeding 1 × 10^4^ cells/well in 96-well plates in complete medium. The day after, fresh medium containing 2% FBS and 1 µg/mL doxycycline hyclate (Sigma Aldrich/Merck, Darmstadt, Germany) was added. Cell viability was determined after 72 h using an MTT [3-(4,5-dimethylthiazol-2-yl)-2,5-diphenyl-2H-tetrazolium bromide Sigma Aldrich, St. Louis, MO, USA] assay, which is based on the conversion of MTT to formazan by mitochondrial enzymes. The formazan deposits were dissolved in dimethyl sulfoxide (DMSO) and the absorbance of each well was measured at 540 nm in a FLUOstar Omega (BMG Labtech, Ortenberg, Germany) Plate Reader (version: 1.30.101.8). Each experimental condition was evaluated with 24 replicates and the experiments were repeated three times. Obtained data were analyzed using GraphPad Prism 10 software.

### 2.6. Cell Cycle Analysis

MCF-7-tRF3E, MCF-7-M19–24 and MCF-7 control cells were seeded onto 6-well tissue culture plates, 5 × 10^5^ cells per well, in complete medium. The day after, cells were starved for 24 h and then treated with 1 µg/mL doxycycline hyclate for an additional 48 h. After 48 h incubation, the cells were harvested and fixed with ice-cold 70% ethanol for 1 h at 4 °C. RNA was digested by 1 mg/mL bovine RNase (Sigma Aldrich/Merck, Darmstadt, Germany) 30 min at 37 °C with shaking. Cells were then labeled with 15 mg/mL propidium iodide (PI) 30 min at 37 °C in the dark. Samples were analyzed by fluorescence-activated cell sorting (FACS) (BD FACScalibur™, BD Biosciences, Becton Dickinson and company, Franklin Lakes, NJ, USA) and data were elaborated via FlowJo software (v 8.7).

### 2.7. Growth Curve Analysis

MCF-7-tRF3E, MCF-7-M19–24 and MCF-7 control cells were plated in 6-well plates in duplicate (4 × 10^4^ cells/well) in a complete medium and treated or not with 1 µg/mL doxycycline hyclate. The cell number was counted on day 2, day 5 and day 7, when cells were detached with Trypsin–EDTA and counted using Trypan blue to exclude dead cells.

### 2.8. Colony Assay

MCF-7-tRF3E, MCF-7-M19–24 and MCF-7 control cells were seeded at the density 600 cells/well in a 6-well plate and incubated in complete medium, with or without 1 µg/mL doxycycline hyclate. After two weeks, plates were stained with crystal violet and colonies (≥50 cells) were counted, as described elsewhere [30]. The surviving fraction was calculated as the ratio of the number of colonies in the treated sample to the number of colonies in the untreated sample.

### 2.9. Western Blot

Cells were homogenized in RIPA buffer (0.1% SDS, 1% NP40, 0.5% CHAPS) supplemented with protease inhibitors (Sigma Aldrich/Merck, Darmstadt, Germany). For Western blotting analysis, equal amounts of protein lysates were separated onto Criterion™ TGX™ precast gels (Bio-Rad, Hercules, CA, USA) and transferred to polyvinylidene difluoride (PVDF) membranes (Immobilion P, Millipore, Burlington, MA, USA) using Criterion™ Blotter (Bio-Rad, Hercules, CA, USA). Membranes were blocked with EveryBlot Blocking Buffer (Bio-Rad, Hercules, CA, USA) and then incubated overnight with primary antibodies at 4 °C. Primary antibodies to NCL (C23 (MS-3); sc-8031, lot#E2023) and β-actin (C4; sc-47778, lot#K1617) were from Santa Cruz Biotechnology (Dallas, TX, USA); the primary antibody to p27 was from Cell Signaling Technology (Danvers, MA, USA) (Cat#3686S). Secondary antibodies conjugated with peroxidase were from Sigma-Aldrich (Darmstadt, Germany). Secondary antibody binding was performed at room temperature for 1 h. After TBS-T washing, membranes were incubated with PierceTM ECL Western Blotting Substrate (Thermo Fisher scientific, Waltham, MA, USA) and the immunoreactive proteins were detected with the ChemiDoc™ XRS-System (Bio-Rad, Hercules, CA, USA). Densitometry analysis was performed using ImageJ software (2.1.0/1.53c).

### 2.10. Immunofluorescence Analysis

MCF-7-tRF3E, MCF-7-M19–24 and MCF-7 control cells were seeded in a 24-well plate (2 × 10^5^ cells/well). After a 48 h incubation with 1 µg/mL doxycycline hyclate, cells were fixed for 10 min with 4% paraformaldehyde (Sigma Aldrich/Merck, Darmstadt, Germany) in phosphate-buffered saline (PBS). After incubation in blocking buffer (PBS, 10% bovine serum albumin (BSA; Sigma Aldrich/Merck, Darmstadt, Germany)) for 20 min, cells were incubated for 1 h at 37 °C with the primary antibody against NCL (mouse monoclonal antibody C23 (MS-3); sc-8031, lot#E2023; Santa Cruz Biotechnology (Dallas, TX, USA); diluted 1:100). After washing, cells were incubated with anti-mouse IgG Alex Fluor 488^TM^ secondary antibody (Invitrogen Molecular Probes, Eugene, OR, USA) diluted at 1:200 for 1 h at 37 °C. Cells were then examined under a fluorescence microscope (Carl Zeiss GmbH, Oberkochen, Germany).

### 2.11. Computational Modeling

The complete structure of NCL was obtained using the model reconstructed according to the protocol already published [26]. Starting from their nucleotide sequences, the 3D RNA structures were modelled using SimRNA v.2.0 (https://genesilico.pl/SimRNAweb) (https://doi.org/10.1093/nar/gkae356 accessed on 28 March 2022). These computational tools rely on the Monte Carlo method for sampling the conformational space and employ a statistical potential to approximate the energy, thereby identifying conformations that correspond to biologically relevant structures. Moreover, the 3D RNA server [31] was used as a second control, confirming the obtained results. The 3D structures of the RNA-NCL complexes were predicted using HADDOCK 2.4 (https://wenmr.science.uu.nl/ accessed on 18 April 2022) (High Ambiguity Driven DOCKing) [32], which has demonstrated reliability as well as flexibility [32,33,34,35]. HADDOCK distinguishes itself from ab initio docking methods by incorporating information from known or predicted protein interfaces into ambiguous interaction restraints (AIRs) to guide the docking process. RNA/NCL complexes were put in a simulation box of 15 × 15 × 15 nm, adding TIP3P water molecules and NaCl, to reach the physiological conditions, taking into account the net charge of NCL protein and RNA molecules. CHARMM-GUI was used for setting the simulation conditions [36]. The systems were minimized with 10,000 cycles steepest descent (SD) followed by 5000 steps conjugate gradient (CG), obtaining a convergence of maximum force to the energy threshold of 1000 kJ/mol nm^2^. Then, 6 equilibration steps let NCL gradually accommodate within the aqueous environment; the Verlet cutoff scheme for neighbor searching, combined with PME for electrostatics, was used. The cutoff for the Van der Waals forces calculation was set to 1.2 nm with force smoothly switched to zero (between 1.0 and 1.2 nm) generating the velocities at 310 K in the NVT ensemble using a Maxwell distribution function with random seed (Berendsen thermostat) (2 simulation runs, 25 ps). Then, we shifted to the NPT ensemble, maintaining the weak coupling also for pressure control (Berendsen barostat, isotropic conditions, 1 bar, time coupling 5 ps), maintained for 4 equilibration runs (50 ps). In the 100 ns production phase, we shifted to Nosé–Hoover and Parrinello–Rahman algorithms for temperature control and pressure coupling, respectively; a leapfrog algorithm and a time step of 0.002 ps were used. On the obtained trajectories, we calculated the MM-PBSA energies of all NCL-RNA systems using the gmx_MMPBSA gromacs tool [37]. The Charmm36m force field was used within the GROMACS 2024.4 software package (https://doi.org/10.5281/zenodo.10589697 accessed on 19 May 2023) [38].

### 2.12. Statistical Analysis

The significance of differences was evaluated with an unpaired Student *t* test when two groups were compared, while a 2-way ANOVA test followed by Šídák’s multiple comparisons test or Tukey’s multiple comparisons test was used to compare three or more groups. Statistical analysis was carried out with GraphPad Prism 10. Differences were considered significant at *p* < 0.05.

## 3. Results and Discussion

### 3.1. Identification of the Molecular Determinants of tRF3E-NCL Interaction

The ability of RNA molecules to bind NCL relies on the presence of specific NCL recognition elements (NREs). NREs, displaying a high binding affinity, have been identified by a selection-amplification protocol (SELEX) and are constituted by an 18-nucleotide (18-nt) exposing the single-stranded 6-nucleotide (6-nt) motif UCCCGA [4]. The tRF3E sequence contains two 6-nt stretches, located at positions 6–11 and 19–24, which exactly match NRE and might be relevant determinants that mediate the RNA-NCL interaction. Thus, to validate this hypothesis, first we confirmed the interaction of NREs with NCL using molecular docking, then we designed two RNA oligonucleotides carrying four-base substitutions on these signature motifs, at positions 7–10 or 20–23 on the tRF3E sequence, to further evaluate the contribution of each consensus motif to NCL binding. These mutated forms of tRF3E were named M6–11 and M19–24, respectively. In detail, we replaced the core CCCG motif with AAAU to disrupt the potential NCL recognition. In addition, we constructed a double mutant, called D2M, bearing the same mutations on both NCL target consensus sequences (6–11 and 19–24) (Figure 1A). Electrophoretic Mobility Shift Assays (EMSAs) were performed to estimate the binding affinity of NCL for the wild-type (wt) tRF3E and its mutants (M6–11, M19–24 and D2M). Unexpectedly, the 19–24 mutation seemed not to affect the interaction with NCL and both tRF3E and M19–24 exhibited a similar dissociation constant (K_D_) of ≅280 nM (Figure 1B).

This K_D_ value is higher than that previously determined by [26] because of the different experimental conditions (incubation temperature and RNA concentration) used in the EMSAs of the present study. However, a binding affinity of ≅280 nM implies a very stable RNA-NCL interaction by virtue of the fact that the K_D_ for natural RNA targets varies from 500 to 1000 nM. Notably, lower K_D_ values (≤50 nM) have been obtained only with in vitro selected sequences [4]. Unlike tRF3E and M19–24, NCL lost completely its ability to retard the M6–11 mutant, whereas it was still able to interact with the double mutant D2M, although with a very low affinity (K_D_ ≥ 1500 nM). All together, these outcomes outline the importance of the 6–11 motif and clearly indicate that mutations in this region and/or modifications of RNA structure, associated with sequence changes, negatively impact the tRF3E-NCL interaction.

**Figure 2:**
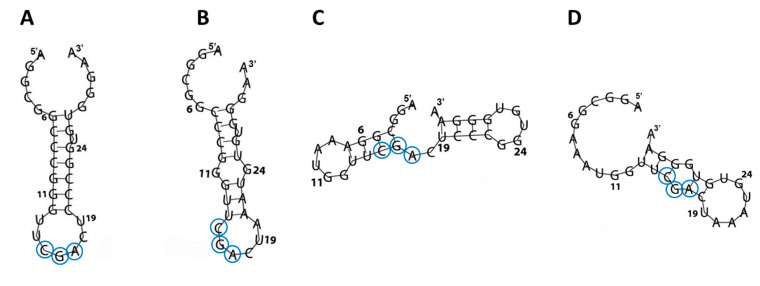
RNA folding of wt and mutated tRF3E. The secondary structures of tRF3E (**A**), M19–24 (**B**), M6–11 (**C**) and D2M (**D**), as predicted by RNAfold (http://rna.tbi.univie.ac.at/cgi-bin/RNAWebSuite/RNAfold.cgi accessed on 7 June 2023), are shown. Nucleotides corresponding to the minimal nucleolin recognition element (CGA), localized in a stem–loop context, are circled.

To correlate the secondary and tertiary RNA structure with the NCL binding properties, the RNA folding of tRF3E and its mutants was predicted in silico by RNAfold (Figure 2) and SimRNA v.2.0 (Supplementary Figure S1). These computational analyses revealed that, diversely from previous studies [4,5], which found the NCL conserved recognition sequence (U/G)CCCG(A/G) within a hairpin loop, the two tRF3E motifs 6–11 and 19–24 are instead located in base-paired regions (stems), proposed to be an adverse condition for NCL binding. Surprisingly, tRF3E and M19–24, in addition to exhibiting a similar three-dimensional (3D) conformation (Supplementary Figure S1), expose the conserved 3-nt CGA (positions 15–17) within a loop that may function as minimal NRE (Figure 2A,B). The relevant role of the 3-nt CGA is supported by NCL gel shift assays carried out with NRE variants carrying mutations in the conserved stem–loop motif. In fact, Johansson et al. [5] showed that in vitro selected NREs containing the G in the first position combined to the G → A substitution in the fifth position (resulting mutated motif: GCCCAA) and almost completely abolished the RNA-NCL interaction (K_D_ ≥ 15 µM). Thus, structural homology and proper arrangement of the 3-nt CGA in tRF3E and M19–24 may explain the comparable binding affinity of NCL to these RNAs. In this respect, the D2M and M6–11 mutants show a distinct arrangement of the CGA motif; in D2M, it is located within an RNA bubble, whereas in M6–11, it lies at the junction between a double-stranded and an unstructured region (Figure 2C,D). Consistently, the residual binding specificity (K_D_ ≥ 1500 nM) of D2M, which indicates a very weak interaction with NCL, and the complete loss of the ability of M6–11 to bind NCL, could reflect profound modifications in the secondary and tertiary structures of these mutants compared to tRF3E. Thus, an unfavorable conformation and the lack of exposure of the 3-nt sequence CGA within a loop may be responsible for abolishing the recognition of M6–11 and D2M by NCL. Docking predictions, carried out with the M6–11 and D2M mutants, confirmed that they were not able to fully interact with RBD1–2 or RBD3–4; both mutants marginally contacted only the RBD2 domain, leaving free RBD3 and RBD4 (Supplementary Figure S2). For these reasons, M6–11 and D2M were not further investigated in this study.

### 3.2. NCL Cooperatively Binds tRF3E

**Figure 3:**
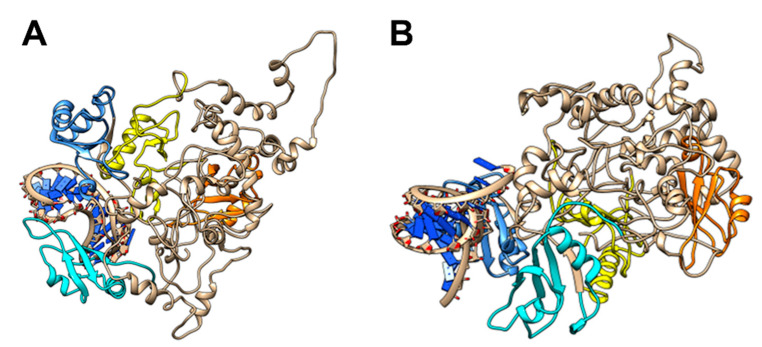
Molecular modelling of NCL with M19–24. The lowest energy models for M19–24 NCL complexes (**A**,**B**) as predicted by HADDOCK 2.4 (see Section 2) are shown. The RBD1 is in yellow, the RBD2 is in orange, the RBD3 is in cyan, the RBD4 is in cornflower blue and RNAs are in dark blue.

Recently, it has been reported that cooperativity among multiple domains of RNA-binding proteins can markedly enhance their intrinsic affinity for RNA targets with respect to that determined for individual domains [39]. We have previously shown that the four RNA-binding domains (RBD1–2 and RBD3–4) can function in pairs, providing evidence that they can be simultaneously occupied by two tRF3E molecules [26]. However, operating with an excess of NCL, the experimental conditions used in the EMSA of Figure 1B, only one of the two RNA binding sites of the protein is bound by tRF3E or M19–24. Indeed, at protein concentrations ranging from 0.4 to 0.7 µM (6–10 pmol), NCL was able to completely capture 2 pmol of tRF3E or M19–24, causing the progressive disappearance of bands corresponding to the free radiolabeled (hot) RNA. Under these experimental conditions and according to previous computational analysis [26], we assumed that only the higher affinity NCL RBD1–2 participated in tRF3E binding giving rise to the RNA-NCL complex I. A retarded band having a similar electrophoretic mobility of complex I was formed also with M19–24 (Figure 1B). Importantly, NCL-docked models predicted for the M19–24 showed that this mutant, unlike the wt tRF3E, was able to sit exclusively in the cleft between RBD3 and RBD4, leaving the RBD1–2 totally free (Figure 3). These findings suggest that the NCL sites 1–2 and 3–4 were not functionally equivalent and the formation of the two distinct complexes, tRF3E-RBD1–2 and M19–24-RBD3–4, is strongly influenced by the presence of native versus mutated NCL target motifs. 

**Figure 4:**
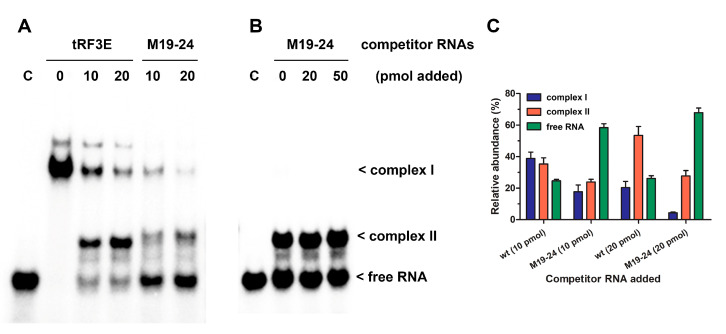
Competitive EMSA of wt tRF3E and M19–24 with NCL. Competitive EMSAs were carried out essentially as described in Figure 1. After a preincubation of 2 pmol (**A**) and 20 pmol (**B**) of [^32^P]-labeled wt tRF3E with 7 pmol (0.5 µM) of NCL, the indicated amounts of not-labeled (cold) tRF3E and M19–24 were added and incubation prolonged. The sample without competition is marked with “0” whereas “C” represents the control in absence of protein. The electrophoretic migration of free RNA and RNA-NCL complexes I and II are indicated. Radioactive signals associated to complex I (blue bars), complex II (red bars) and free RNA (green bars) were quantified by imager and expressed as a percentage of total radioactivity per each lane (**C**). Values are means ± SEM obtained from panel (**A**) and EMSA reported in Supplementary Figure S3.

To gain deeper insight into the RNA-NCL interaction, we carried out EMSAs in which the pre-formed tRF3E-NCL complex was challenged by increasing concentrations of either wt tRF3E or M19–24. As expected, with an excess of NCL (7 pmol of protein versus 2 pmol of hot tRF3E) and in the absence of any competitor RNA, only the complex I was formed (Figure 4A, lane 0). Upon the addition of unlabeled (cold) tRF3E, complex I drastically dissociated to generate complex II, which represented 60% of total radioactivity at 20 pmol of cold wt tRF3E as competitor (Figure 4A,C). In detail, according to the proposed model, increased amounts of cold tRF3E, instead of displacing the hot tRF3E, already bound to RBD1–2, saturated both RBD1–2 and RBD3–4, originating the NCL complex II. Indeed, upon the addition of 20 pmol of cold tRF3E, corresponding to a 10-fold higher amount of hot tRF3E (2 pmol), only 25% of RNA was recovered in its free form. Differently, when the tRF3E-NCL complex I was challenged with an excess (10 and 20 pmol) of cold M19–24 as competitor, it quickly disappeared, giving rise to a low quantity of complex II (24–28%) and consequently most of the hot probe (58–68%) was released as free RNA (Figure 4A,C). These results suggest that the mixed complex II, in which tRF3E and M19–24 are respectively bound to RBD1–2 and RBD3–4, is transient and extremely less stable than the native complex II, containing two wt tRF3E molecules. Accordingly, molecular dynamics study (Section 3.3) revealed that the interaction of M19–24 with RBD3–4 leads to a consistent change in the 3D structure of the overall RNA-NCL complex, which in turn negatively impacts the binding property also of RBD1–2 for its target RNA. As a result, wt tRF3E is kicked out from RBD1–2 and the spurious complex II dissociates. In other words, the M19–24-NCL interaction causes the loss of cooperativity between the two RNA-binding domains (RBD1–2 and RBD3–4).

**Figure 5:**
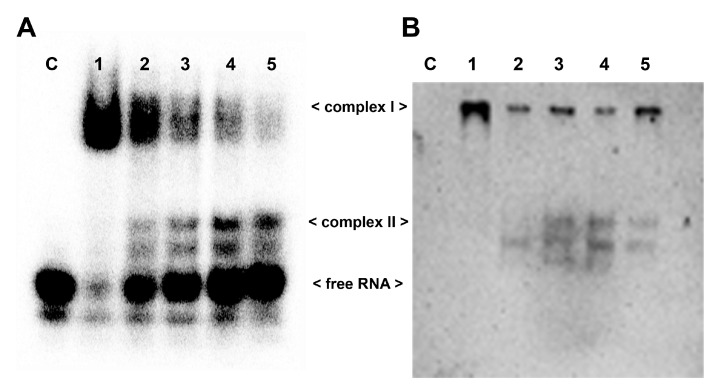
Identification of tRF3E-NCL complexes by EMSA-Western blotting analysis. (**A**). EMSA was carried out as described in Figure 4A with 2 pmol of [^32^P]-labeled wt tRF3E and 7 pmol (0.5 µM) of NCL. “C” represents the control in absence of protein whereas samples from 1 to 5 contain 0, 5, 10, 20 and 30 pmol of not-labeled wt tRF3E as competitor, respectively. (**B**). After running, the polyacrylamide gel (panel A) was incubated in a 1% SDS denaturing solution for 2 h and blotted on a PVDF membrane as described in Section 2. The NCL protein in complex I and complex II was visualized thanks to the peroxidase-conjugated secondary antibody directed against the anti-His tag primary antibody, recognizing NCL.

Undeniably, RNA molecules within cells are in excess and NCL utilizes at one time both RBD1–2 and RBD3–4 to interact with NREs on its natural targets, including messenger RNAs. To more closely mimic this condition, we carried out an EMSA using an overload of wt tRF3E (20 pmol of RNA versus 7 pmol of protein) that drastically triggers the formation of the only NCL complex II, rather than both complexes. As seen in Figure 4B, the complex II, bearing two wt tRF3E molecules bound respectively to NCL sites 1–2 and 3–4 (tRF3E-RBD1–2·RBD3–4-tRF3E), did not dissociate upon addition of increasing amounts of cold M19–24 (20 and 50 pmol). Indeed, the intensity of radioactive signals associated with the retardation band of complex II remained constant as well as the free RNA. Taken together, these findings indicate that the native tRF3E-NCL complex II is somehow “locked” and the mutant M19–24, unlike what was observed in complex I, is unable to displace wt tRF3E even from RBD3–4. Notably, the RNA-NCL complex II exhibited a higher electrophoretic mobility than complex I. It has been well established that migration of DNA- and RNA-protein complexes, on native gels under constant-field conditions, is primarily dependent on the negative charges of nucleic acids. Consequently, complex II in which NCL is bound to two tRF3E molecules moves faster than complex I, containing only one RNA molecule. To definitely prove that both complex I and II arose from the tRF3E-NCL interaction, the competitive EMSA was subjected to Western blotting and NCL was immunologically detected. As clearly shown, there is an almost perfect correspondence between the retarded bands obtained in the EMSA (Figure 5A) and the protein localization revealed by anti-NCL antibody (Figure 5B).

### 3.3. Molecular Dynamics of tRF3E and M19–24 NCL Complexes

Findings obtained with tRF3E allowed us to have an insight into the key determinants governing the dynamic of RNA-NCL interaction and possibly open the way for a deeper comprehension of how NCL recognizes its target RNAs and the regulatory implications of this process. The formulated model predicts that the NCL RBD1–2 and RBD3–4 do not function in the same manner. RBDs show a high degree of cooperativity only with wt tRF3E and respond differently to native or mutated NREs present on the tested RNAs. A previous in silico study revealed that wt tRF3E strongly interacts with NCL RBD1–2, whereas its binding to RBD3–4 is less favorite. Thus, when two tRF3E molecules simultaneously filled up both binding sites, the tRF3E-NCL-tRF3E complex II underwent a drastic stabilization, as validated by a 2.6-fold reduction in the complex binding energy compared to a system containing only one tRF3E molecule bound to RBD1–2 [26].

**Figure 6:**
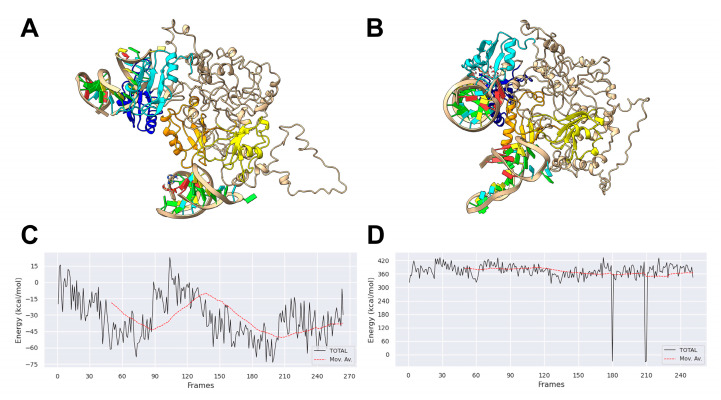
Molecular modelling of NCL with tRF3E and M19–24. The lowest energy models for the tRF3E-RBD1–2·RBD3–4-tRF3E complex (**A**) and for the mixed tRF3E-RBD1–2·RBD3–4-M19–24 complex (**B**) as predicted by HADDOCK 2.4 (see Section 2) are shown. The RBD1 is in orange, the RBD2 is in yellow, the RBD3 is in cyan, the RBD4 is in cornflower blue and RNA nucleotides are represented in Tube/Slab representation using red for A, cyan for U, green for G and yellow for C. The binding free energy was calculated using the gmx_MMPBSA gromacs tool (see Section 2) for the tRF3E-RBD1–2-NCL-RBD3–4-tRF3E complex (**C**) and for the mixed complex tRF3E-RBD1–2·RBD3–4-M19–24 (**D**). The simulation time was 100 ns.

Here, in order to better rationalize the experimental results, we compared by molecular dynamics simulations the stability of a native complex II (tRF3E-RBD1–2·RBD3–4-tRF3E) with that of a mixed one, containing tRF3E in RBD1–2 and M19–24 in RBD3–4 (tRF3E-RBD1–2·RBD3–4-M19–24) in their predicted bound state. As evident in Figure 6A,B, the overall 3D structures of the two RNA-NCL complexes differ significantly, involving modifications of the spatial organization of all four NCL RBDs. These changes affected the stability of the complex, which was greater for tRF3E-RBD1–2·RBD3–4-tRF3E (ΔG_complex_ ≅ −22,000 kcal/mol) than for tRF3E-RBD1–2·RBD3–4-M19–24 (ΔG_complex_ ≅ −20,000 kcal/mol) (Table 1). In addition, the spurious complex exhibited a remarkably positive value of the binding free energy (+361 ≤ ΔG_binding (kcal/mol)_ ≤ +371) (Figure 6D and Table 1). It is noteworthy that ΔG_binding_ expresses the difference in free energy between the bound and completely unbound states and positive values indicate an unfavorable interaction. Thus, the tRF3E-RBD1–2·RBD3–4-M19–24 complex is destabilized and prone to suddenly dissociate, releasing both tRF3E and M19–24 from the respective binding sites as evidenced by the appearance of a very intense band (≅70% of total radioactivity) corresponding to free RNA in the EMSA (Figure 4A,C). Conversely, ΔG_binding_ for the tRF3E-RBD1–2·RBD3–4-tRF3E complex was negative (from −32.88 to −41.21 kcal/mol), suggesting a strong RNA-NCL interaction (Figure 6C and Table 1).

Molecular dynamics simulation fully supported the data from band shift assays, providing precious information on the kinetics of occupancy of the four NCL RBDs, RNA–protein 3D structures and the binding energies of resulting complexes.

In addition to its identified substrate (NRE) [4,5], NCL has been shown to bind also with a very high affinity to (G)-rich sequences. These DNA or RNA regions are capable of folding into highly stable four-stranded secondary structures known as G-quadruplexes (G4s), which display specific regulatory roles in different cellular processes [17,40,41]. Given that tRF3E contains five sequential runs of Gs, a canonical feature of G4, its potential to adopt a G-quadruplex conformation was assessed using the G4RNA Screener tool (http://scottgroup.med.usherbrooke.ca/G4RNA_screener/ accessed on 8 July 2024). This in silico analysis revealed that the tRF3E molecule has a moderate propensity of rearranging into a G4 secondary structure as reflected by consistently low predictive scores (consecutive G over consecutive C ratio (cGcC), 1.4783; G4Hunter (G4H), 0.3438; G4 Neural Network (G4NN), 0.0644). Notably, when the score was calculated for a well-known DNA sequence (36-nt oligonucleotide from human telomeres) able to form G-quadruplex [42], the values were extremely higher than those obtained for tRF3E (cGcC = 1.50, G4H = 600 and G4NN = 0.9984). Therefore, we cannot exclude that the interaction between NCL and tRF3E might rely on alternative higher-order RNA configurations, but it does not seem to be mediated by the canonical G4 formation.

### 3.4. The Occupancy of Both NCL RBD1–2 and RBD3–4 Is Required for tRF3E Antitumor Function in Breast Cancer Cells

**Figure 7:**
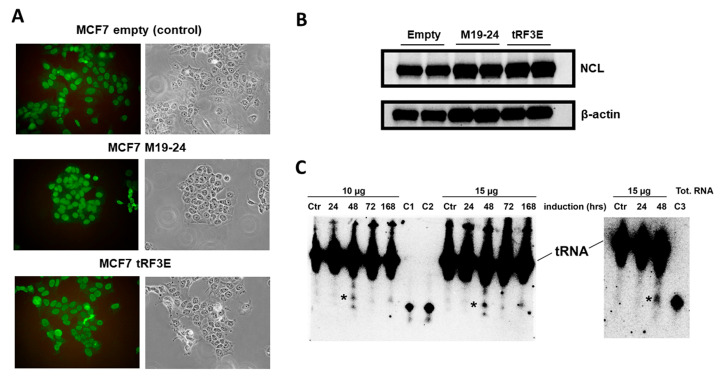
NCL expression and tRF3E or M19–24 induction in MCF-7 cell lines. (**A**). Immuno-localization of NCL in MCF-7-tRF3E, MCF-7-M19–24 and MCF-7 control cells. Cells were processed for immunofluorescence with anti-NCL antibody and then observed with a fluorescence microscope (Carl Zeiss GmbH, Germany). (**B**). Representative Western blot showing the expression of NCL and β-actin (loading control) in MCF-7-tRF3E, MCF-7-M19–24 and MCF-7 control cells. Samples in duplicate containing 20 µg of proteins/well were loaded. (**C**). Northern blotting analysis of wt tRF3E (left gel) and M19–24 (right gel) was performed on total RNA (10 µg and 15 µg), extracted from MCF-7-tRF3E cells left untreated (Ctr) or treated with 1 µg/mL doxycycline for the indicated times to induce tRF3E and M19–24 (marked with asterisks). Total RNA was run on a denaturing 8% PAGE-urea 7M gel and transferred by electroblotting to nylon membranes that were hybridized with a [^32^P]-labeled tRF3E probe. As standards, known quantities of synthetic RNAs were loaded as follows: C1 and C2 contained 0.3 ng and 0.6 ng of tRF3E whereas C3 contained 0.6 ng of M19–24.

To clarify the functional role of tRF3E in breast cancer, SK-BR-3 and MCF-7 breast cancer cell lines were transiently transfected with tRF3E and its ability to reduce cancer cell viability was assessed by MTT assay (Supplementary Figure S4). To corroborate tRF3E’s tumor suppressive function, MCF-7 cells were stably transduced with a pLKO-Tet-On vector properly engineered in order to allow the expression of tRF3E upon doxycycline induction; they were called MCF-7-tRF3E cells. In parallel, MCF-7 cells were stably transduced with the empty vector and used as a control (MCF-7 control cells). Moreover, a third MCF-7 transduced cell line was generated using a pLKO-Tet-On vector encoding the M19–24 mutant (MCF-7-M19–24 cells) that, according to in silico analysis and EMSA results, binds NCL on RBD3–4 but not on RBD1–2. Because of that, MCF-7-M19–24 cells permitted evaluation of the functional consequences of the loss of a cooperative interaction between tRF3E and NCL. MCF-7-tRF3E, MCF-7-M19–24 and MCF-7 control cells expressed similar levels of NCL, localized mainly in nuclei, as expected (Figure 7A,B). A Northern blotting analysis was performed to verify the expression of wt tRF3E and its mutated form M19–24 in MCF-7 cells upon doxycycline induction, and thus to validate this new in vitro model. As shown in Figure 7C, the expression of wt tRF3E and M19–24 starts to be clearly detectable after 48 h of doxycycline induction.

**Figure 8:**
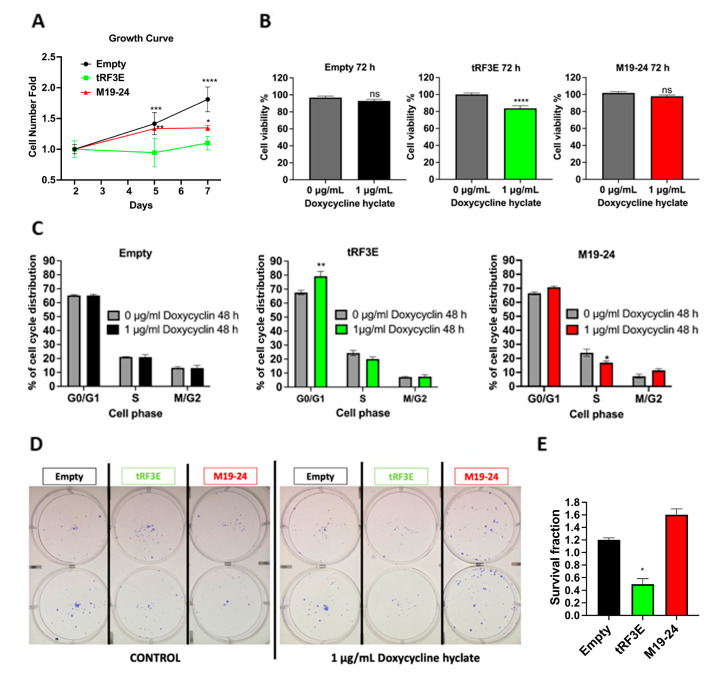
Functional characterization of tRF3E-expressing MCF-7 cells. (**A**). Growth curve. MCF-7 control cells (empty) and MCF-7-tRF3E cells and MCF-7-M19–24 cells were seeded in 6-well plates (40,000 cells/well) and treated with 1 µg/mL doxycycline. The cell number was counted on day 2, day 5 and day 7. The graph shows cell number fold change with respect to day 2 (*n* = 4). Values are means ± SD. Two-way ANOVA followed by Tukey’s multiple comparisons test. * *p* < 0.05, ** *p* < 0.01, *** *p* = 0.0001, **** *p* < 0.0001 (MCF-7-tRF3E versus (vs.) MCF-7 control cells or vs. MCF-7-M19–24 cells). (**B**). Effect of tRF3E expression on MCF-7 cell viability determined by MTT assay. MCF-7 control cells (empty) or MCF-7-tRF3E cells or MCF-7-M19–24 cells were left untreated (grey) or induced with 1 µg/mL doxycycline for 72 h, to express tRF3E (green), or M19–24 (red) or any tRF (black). The results are expressed as percentage of viable cells with respect to control. Values are means ± SEM (*n* = 20). Unpaired *t* test. **** *p* < 0.0001. (**C**). Cell cycle analysis by FACS. Histograms show the percentage of MCF-7 control cells (empty) or MCF-7-tRF3E cells or MCF-7-M19–24 cells in G0/G1, S and G2/M phases, when they are not induced (grey) or induced with 1 µg/mL doxycycline for 48 h. Data are presented as the mean ± SEM of three repeats. Two-way ANOVA test followed by Šídák’s multiple comparisons test. * *p* < 0.05; ** *p* < 0.005. (**D**). Colony assay of MCF-7 control cells (empty; black), MCF-7-tRF3E cells (green) and MCF-7-M19–24 cells (red) plated at low density (600 cell/well) and left untreated (control) or induced with 1 µg/mL doxycycline for 2 weeks. Colonies were stained with crystal violet and counted. Representative images from two independent experiments, performed in duplicate. (**E**). Data from panel D are expressed as survival fraction and the mean ± SEM is shown. Statistical significance was calculated using the unpaired *t* test. * *p* < 0.05 (tRF3E vs. empty); M19–24 vs. Empty: not statistically significant.

Then, the three MCF-7 transduced cell lines were characterized evaluating their proliferation rate, viability and clonogenicity upon doxycycline treatment. In particular, to compare the proliferation rates of these cell lines, a growth curve was performed by plating equal numbers of cells, at low density, in a 6-well plate in the presence of doxycycline. Cells were then collected and counted after 2, 5 and 7 days. The obtained growth curves indicate that cells expressing tRF3E exhibit a statistically significant lower proliferation rate compared to the other two cell lines (Figure 8A). These results are consistent with previous findings demonstrating the inhibitory effect of tRF3E on cell proliferation [26]. Moreover, the induction of tRF3E expression by a 72 h treatment with doxycycline caused a 20% reduction in MCF-7-tRF3E cell viability, as assessed by an MTT assay. In contrast, the viability of both control and doxycycline-induced MCF-7-M19–24 cells remained unaffected, suggesting that M19–24 lacks the functional properties of wt tRF3E (Figure 8B). Next, we examined the impact of tRF3E and M19–24 expression on cell cycle distribution by flow cytometry analysis. As expected, doxycycline treatment did not alter the percentage of MCF-7 control cells in G0/G1 phase, as well as in S or in M/G2 phases. On the contrary, induction of tRF3E resulted in a statistically significant increase in the percentage of MCF-7-tRF3E cells in the G0/G1 phase (79.2 ± 6%) with respect to uninduced cells (67.6 ± 2.5%), while the proportion of cells in S phase and M/G2 phase remained largely unchanged. The expression of the mutant M19–24 did not significantly shift the percentage of MCF-7-M19–24 cells in G0/G1 phase; however, it seemed to reduce the cell number in S phase and slightly increase the proportion of cells in G2/M phase (Figure 8C).

**Figure 9:**
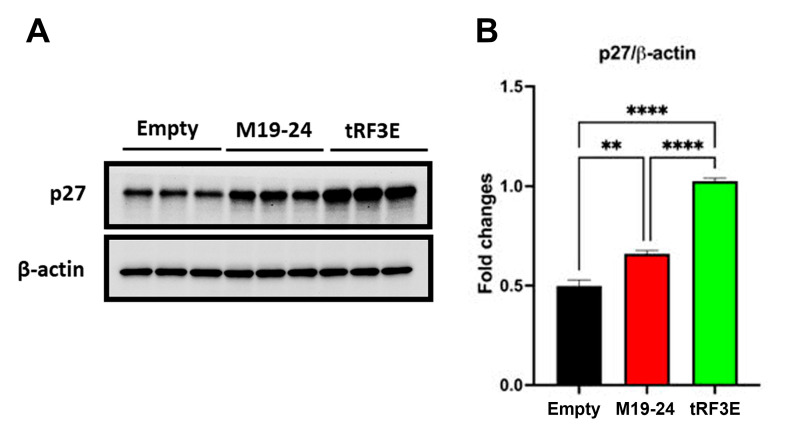
p27 expression increases in tRF3E-expressing MCF-7 cells. (**A**). Representative Western blot showing the expression of p27 and β-actin (loading control) in MCF-7 control cells (empty), MCF-7-M19–24 cells and MCF-7-tRF3E cells treated with 1 µg/mL doxycycline for 48 h. A total of 20 µg of proteins/well were loaded. (**B**). Densitometric quantifications of p27 expression, normalized on β-actin, are shown; data are presented as the mean ± SEM of three repeats. ** *p* < 0.01, **** *p* < 0.0001. One-way ANOVA, followed by Tukey’s multiple comparisons test.

Finally, to further evaluate the functional consequence of tRF3E expression, a colony formation assay was performed by plating cells at low density (600 cells per well in a 6-well plate). This test permits us to assess the ability of residual tumor cells to form recurrences. As shown in Figure 8D,E, a statistically significant reduction in colony number was found in induced MCF-7-tRF3E cells as compared with the control ones. In contrast, no reduction in clonogenicity was observed in MCF-7 cells expressing the M19–24 mutant. Thus, the expression of tRF3E impaired MCF-7 cell survival and their ability to form colonies. In summary, tRF3E is capable of controlling the malignant behavior of breast cancer cells. Notably, tRF3E tumor-suppressor properties are lost upon disruption of the NCL binding motif in position 19–24, suggesting that a cooperative binding involving both RBD1–2 and RBD3–4 domains is required for NCL-mediated tRF3E antitumor functions. Interestingly, we found that the functional ability of tRF3E to control cell proliferation is associated with a significant increase in the level of p27 in MCF-7-tRF3E cells induced for 48 h with doxycycline (Figure 9). p27, encoded by the CDKN1B gene, is a cyclin-dependent kinase (CDK) inhibitor of the kinase-inhibitory protein (Kip) family, which mediates cell-cycle inhibition. In normal cells, p27 levels are tightly regulated across the cell cycle and an increase in p27 can efficiently inhibit G1–S-phase cyclin-CDKs [43,44].

## 4. Conclusions

Breast cancer is the leading cause of cancer-related deaths in women worldwide. A deeper knowledge of the relevant molecular mechanisms behind the onset and progression of BC is needed in order to identify new targets and develop more effective therapies. Nucleolin (NCL) is an RNA-binding protein considered a relevant target in cancer [45]. Indeed, NCL can bind cancer-related mRNAs, controlling their stability and translation, as well as non-coding RNAs. Among NCL-interacting partners, tRNA fragments (tRFs) emerge as crucial NCL modulators, displaying functional roles in post-transcriptional gene regulation [21]. In this study, we analyzed the molecular determinants of RNA-NCL interaction, focusing on the tRF3E-NCL complex. tRF3E is a tumor-suppressor tRF derived from mature tRNA^Glu^, that operates through a mechanism dependent on its physical interaction with NCL. We found that cooperativity among multiple NCL RNA-binding domains (RBD1–2 and RBD3–4) is required for NCL-mediated tRF3E antitumor functions: two tRF3E molecules can simultaneously occupy RBD1–2 and RBD3–4, leading to a drastic stabilization of the tRF3E-NCL complex. Reported results shed light on the dynamic of NCL interaction with its target RNAs and provide crucial information for the development of an RNA-based drug targeting NCL.

## Figures and Tables

**Table 1:** Computational ΔG estimation of RNA-NCL complexes.

	tRF3E-NCL-tRF3E	tRF3E-NCL-M19–24
ΔG_complex_ (complex stability, 100 ns)	−22,165 ± 135 kcal/mol	−20,373 ± 128 kcal/mol
ΔG_complex_ (complex stability, last 50 ns)	−22,224 ± 105 kcal/mol	−20,397 ± 130 kcal/mol
ΔG_binding_ (100 ns)	−32.88 ± 12 kcal/mol	371 ± 50 kcal/mol
ΔG_binding_ (last 50 ns)	−41.21 ± 14 kcal/mol	361 ± 66 kcal/mol

## Data Availability

The data presented in this study are available in this article and in the Supplementary Materials.

## Supplementary Materials

The following supporting information can be downloaded at https://www.mdpi.com/article/10.3390/biom15071054/s1, Figure S1: RNA folding of wild-type and mutated tRF3E. Figure S2: 3D docked complex between NCL and mutated tRF3E M6–11 and D2M. Figure S3: Competitive EMSA of wild-type tRF3E and M19–24 with NCL. Figure S4: tRF3E affects SK-BR-3 and MCF-7 breast cancer cell viability.

## Abbreviations

The following abbreviations are used in this manuscript:

NCL	Nucleolin
RBD	RNA-Binding Domain
EMSA	Electrophoretic Mobility Shift Assay
tRF	tRNA-derived Fragment
