# Supplementary material for: Dissecting the tRNA Fragment tRF3E–Nucleolin Interaction: Implications in Breast Cancer
Source: Biomolecules. 2025 Jul 21;15(7):1054. doi: 10.3390/biom15071054 (PMC12293987; doi:10.3390/biom15071054)
Supplement: Supplementary file 1 [file biomolecules-15-01054-s001.zip › biomolecules-3721356-supplementary.pdf]

SUPPLEMENTARY INFORMATION

Dissecting the tRNA fragment tRF3E-Nucleolin interaction: implications in breast cancer

Maurizio Falconi[1†*], Junbiao Wang[1†], Andrea Costamagna[2], Mara Giangrossi[1], Sunday Segun Alimi[1], Emilia Turco[2], Massimo Bramucci[3], Luana Quassinti[3], Rossana Petrilli[1], Michela Buccioni[3], Gabriella Marucci[3], Augusto Amici[1], Paola Defilippi[2], Roberta Galeazzi[4†], Cristina Marchini[1†*]

[1] *School of Biosciences and Veterinary Medicine, University of Camerino, 62032 Camerino, Italy.*

[2] *Department of Molecular Biotechnology and Health Sciences, University of Turin, 10126 Turin, Italy.*

[3] *School of Pharmacy, University of Camerino, 62032 Camerino, Italy.*

[4] *Department of Life and Environmental Sciences, Marche Polytechnic University, 60131 Ancona, Italy.*

[*] Correspondence: Cristina Marchini, School of Biosciences and Veterinary Medicine, via Gentile III da Varano, University of Camerino, Camerino, 62032, Italy. Tel: +39 0737 403275; Email: cristina.marchini@unicam.it; Maurizio Falconi, School of Biosciences and Veterinary Medicine, via Gentile III da Varano, University of Camerino, Camerino (MC), 62032, Italy. Tel: +39-0737-403274; Email: maurizio.falconi@unicam.it.

[†] These authors contributed equally to this work.

A **B** **C** **D**

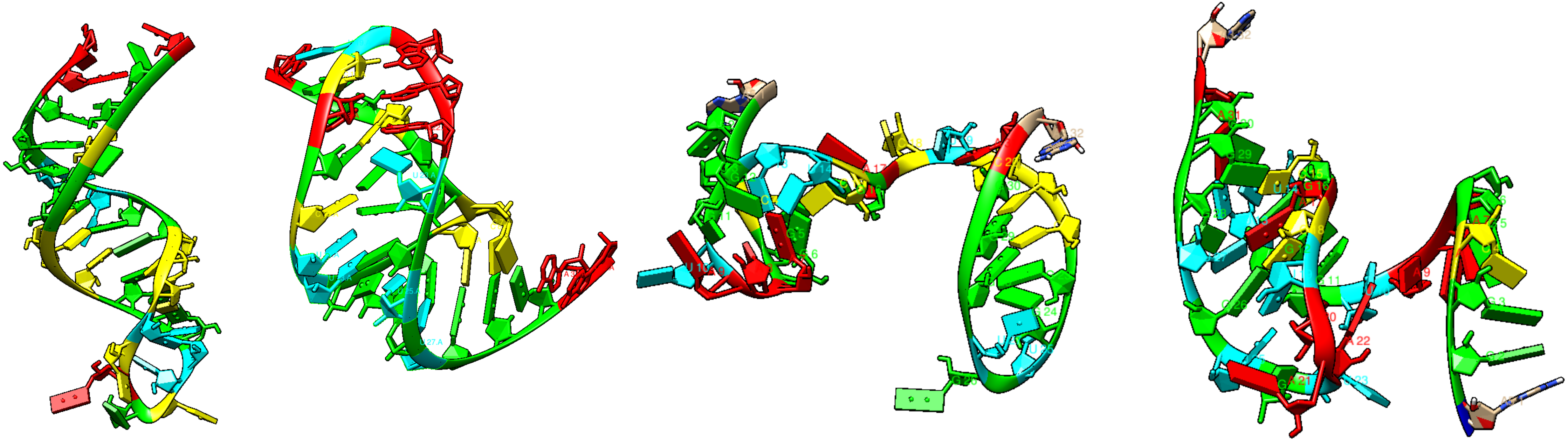

Supplementary Figure S1. RNA folding of wt and mutated tRF3E. The 3D structures of wt tRF3E (**A**), M19-24 (**B**), M6-11 (**C**) and D2M (**D**), as predicted by SimRNA v.2.0 (https://genesilico.pl/SimRNAweb) are shown. Adenine is in red, cytosine is in yellow, guanine is in green and uracil is in blue.

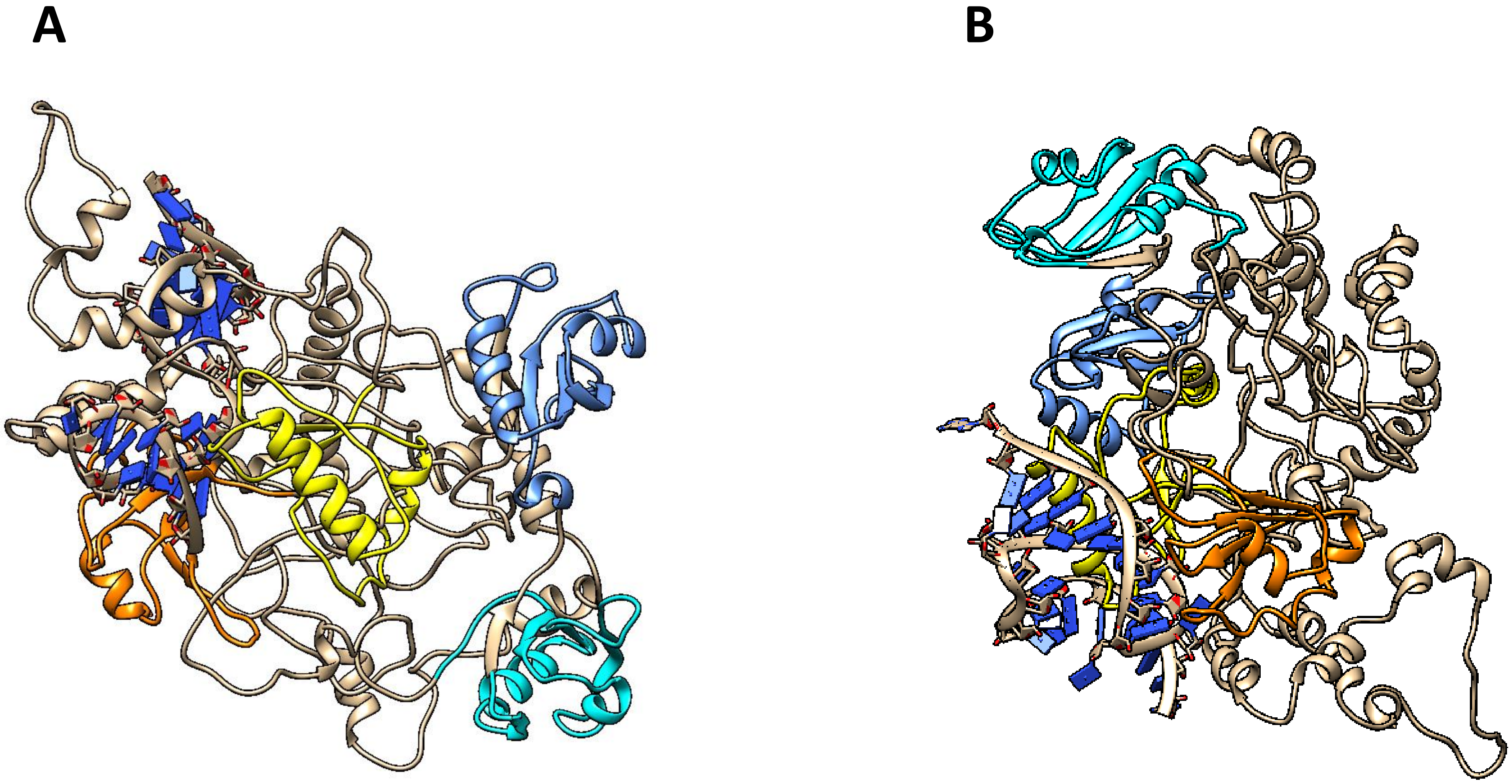

Supplementary Figure S2. 3D docked complex between NCL and mutated tRF3E M6-11 (A) and D2M (B). The protein-RNA docking has been carried out using HADDOCK 2.4 (*Materials and Methods*). Only the lowest energy models are reported. The RBD1 is in yellow, the RBD2 is in orange, the RBD3 is in cyan, the RBD4 is in cornflower blue and the RNA is in dark blue.

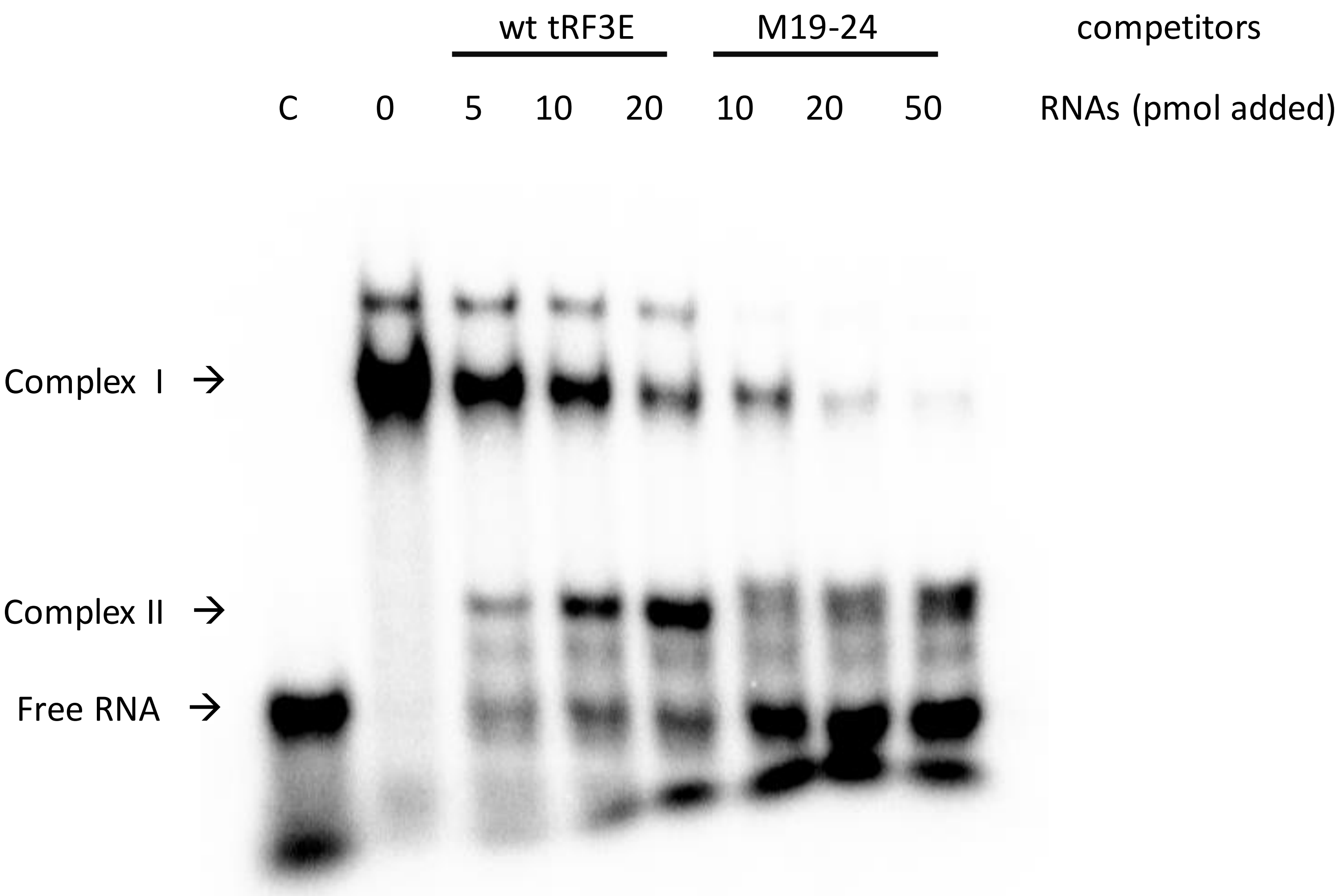

Supplementary Figure S3. Competitive EMSA of wt tRF3E and M19-24 with NCL. Competitive EMSA was carried out essentially as described in **Figures 1** and **4** incubating 2 pmol of [^{32}P]-labeled wt tRF3E with 7 pmol (0.5 µM) of NCL. The amounts of not labeled (cold) wt tRF3E and M19-24 added as competitors are indicated. The sample without competition is marked with “0” whereas “C” represents the control in absence of protein. Bands corresponding to free RNA and RNA-NCL complex I and II are indicated.

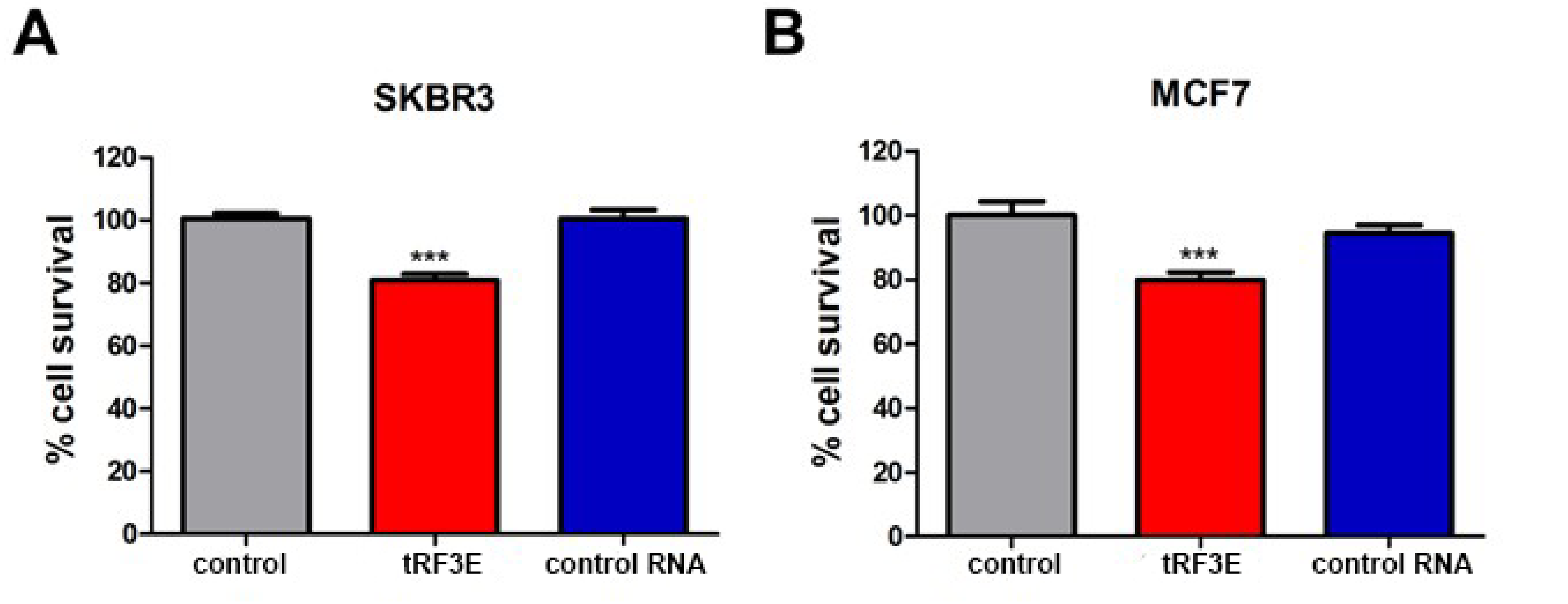

Supplementary Figure S4. tRF3E affects SK-BR-3 and MCF-7 breast cancer cell viability. SK-BR-3 cells (A) and MCF-7 cells (B) were seeded in 96 well plates (1x10^4 cells/well). After 24h, cells were transiently transfected with 200 nM of tRF3E (IDT), or a control RNAs, using Oligofectamine (Invitrogen) as transfection reagent and following manufacturer's instructions and as reported by Falconi et al. (26). The effect of tRF3E on cell viability was evaluated 48 h after transfection by an MTT assay. Results are expressed as percentage of cell viability relative to control cells (treated only with oligofectamine). Data are presented as mean $\pm$ SEM (n=16). The significance was determined by unpaired two-tailed student t test, ***$p < 0.001$ (tRF3E vs control).
